# The X-Rays in Dentistry

**Published:** 1902-08-15

**Authors:** P. M. Hickey

**Affiliations:** Detroit, Mich.


### THE X-RAYS IN DENTISTRY.

BY P. M. HICKEY, M.D., DETROIT, MICH.

Read before the Michigan State Dental Association, June 11, 1902.

When the X-ray or more preferably the Roentgen-ray, was first brought before the scientific world, it was thought that its use would be limited to the investigation of fractures of bone and the detection of foreign metallic substances in the body. However, as thought and time were expended in the investigation of the peculiar properties of this new form of energy, the Roentgen-ray was applied to help in the elucidation of many of the problems of internal medicine. Its application to dentistry was made early in its history and the perfectation of its present technique has been made possible by the work of Harrison, Goldie, Haughton, Price, Collins, Custer and many others.

The uses of the X-ray in dental surgery may perhaps be summarized as follows:

1. To determine the presence of unerupted teeth suspected to be present, or which may be causing neuralgia.

2. To determine the position of roots before beginning the regulation of teeth.

3. To determine the presence, cause and extent of alveolar abscess.

4. To detect fractures of roots or of the maxillary bones by violence.

5. To investigate the root canal to find if it has been filled or if it contains some accidental foreign body.

6. To determine the lower boundry of the antra.

While there are other applications to which the X-ray can be put in dentistry, those which have been enumerated are perhaps the most important. To illustrate the use, I will give briefly the history of a few cases, which to my mind are good examples of the value of the Roentgen-ray.

Case 1. Miss S., age 30, patient of Dr. Shattuck. Some eight or ten years ago the second bicuspid was extracted. Some years later the patient was treated by several practitioners for supposed trouble with the antrum, there being a constant offensive discharge from an opening in the gum just above the first bicuspid. When the patient passed into the hands of Dr. Shattuck, he thought that the source of the trouble was in the space which had been left after extraction of the bicuspid, and accordingly the alveolar process was thoroughly curetted at this point. However, there was no diminution in the nature and character of the discharge. Desiring to elucidate, if possible, the cause of the persistent suppuration, a skiagraph was taken which showed a broken root of the second bicuspid. This was extracted with the happy effect of at once relieving permanently the trouble.

Case 2. Miss C., age 22, patient of Dr. H. C. Corns. Suffered for two years from fistula opening in the upper part of the neck. This had been treated by a half dozen different physicians, with different local applications, such as ointments, washes, etc., the discharge being, however, unchecked and proving a constant source of great annoyance. Dr. Corns on careful investigation came to the conclusion that the fistula extended back to either the first or second molar, the first molar had been crowned some time before and there was a cavity in the crown of the second molar. At the request of Dr. Corns, three skiagraphs of the first and second molars were taken, and in each of them could be seen a delicate shading at the root of the second molar indicating an alveolar abscess at that point. Extraction and local treatment relieved the patient permanently from the annoying discharge and the fistulus opening has now closed.

Case 3. Mr. K., age 14, patient of Dr. J. M. Thompson. Temporary cuspid extracted two months ago. Temporary cuspid had a very long root. As patient should have had the permanent cuspid two years before and as no signs of it appeared on external examination, a skiagraph was made which shows the cuspid in process of descent.

Case 4. M., age 12. The skiagraph in this case showed a lateral incisor to be permanently absent and a missing bicuspid to be unerupted.

It is perhaps unnecessary to enumerate further examples in which the Roentgen-ray can be found of service in dental surgery. It will now be proper to devote a short time to the consideration of the technique of its application. In order to consider this systematically, we may take up first the methods of the production of electrical energy; second, the transferance of this electrical energy into the X-ray and third, the methods of recording the effect of the X-ray on different substances.

There are three methods of obtaining the electricity to energize the Crooke's tube. First, a static machine. Here we have glass plates rapidly revolving by means of a suitable motor furnishing a current which is of high voltage and insignificant amperage. The static machine furnishes a current which is very suitable for fluoroscopic work, but which is inferior for radiographic work, unless the machine be composed of many large plates and these rotated rapidly. The Tesla apparatus has some advocates, but is very destructive to the tubes. Third, the induction coil deriving its energy either from primary or storage batteries or from the electric lighting circuit. These induction coils have been very much improved in their construction within the last few years, and furnish a striking example of the development of an appliance as soon as a need for it exists. It is probable that the induction coil will be further developed within the next few years owing to the greater commercial demands of wireless telegraphy. For dental radiography an eight, ten or twelve-inch coil, operated by current from the electric lighting circuit, is sufficient for all needs. The larger the coil the greater the volume of secondary discharge and consequently shorter exposures. The efficiency of the induction coil depends practically upon the interrupter employed. At first the vibrator interrupter was used. However, with large coils this was found to have many inconveniences. The various forms of electrolytic interrupters have recently been extensively employed; of these I may mention the Wehnelt and the Caldwell.

The Wehnelt interrupter consists of a platinum point and a lead plate immersed in dilute sulphuric acid. When a sufficiently powerful current has passed through the liquid, the platinum and lead being the electrodes, the rapid evolution of gas by the electrolytic action around the platinum point, makes and breaks the current with great frequency.

The Caldwell interrupter is simpler and consists of two lead electrodes immersed in dilute sulphuric acid, one electrode being separated from the other by either a glass or porcelain tube in the wall of which a minute opening exists. The Caldwell interrupter requires less attention then the Wehnelt, as there is no platinum point to wear away. The disadvantages of the electrolytic interrupters are that they are very noisy and give off unpleasant fumes. However, they can be put in separate rooms and thus this inconvenience avoided.

A kind of interrupter which is rapidly coming into favor is the new mercury jet interrupter. In this form a fine stream of mercury, connected with either the positive or negative pole, is forcibly thrown against rapidly rotating pieces of metal connected with the other electric pole. The striking of the mercury jet against the piece of metal makes the current, and the rotation of the metal out of the reach of the jet breaks the current. In this way the interruption of the current is very sharp and the number and length of interruptions are easily variable.

The object of the types of apparatus just spoken of is to furnish a secondary discharge of high voltage. These secondary discharges are sent through an exhausted tube provided with suitable terminals.

### THE GENERATION OF THE X-RAY.

The problem of the conversion of the secondary discharges into the X-ray is perhaps more complex than is its production. We find that glass and metal are oftentimes quickly destroyed by the heavy discharge sent through the Crooke's tube. The most important trouble that we have, beside the finding of a tube heavy enough to withstand these enormous voltages, is to regulate the vacuum in the tube. It is found that the longer a tube is used, especially where the platinum target is allowed to become hot, the higher the vacuum rises. This rise of vacuum is accompanied by increased power of penetration and loss of contrast until finally a point is reached where the vacuum of the tube becomes so high that the current will not pass through the tube and the tube is dead. This point of high vacuum will vary with some tubes with different machines.

A tube, which I think is very efficient for dental work, is the Truax-Greene tube made in Germany, which has a convenient method of lowering the vacuum. This tube permits of a heavy discharge for a short space of time and is consequently suited for dental work, as here the exposures, owing to the slight amount of tissue to be traversed, are particularly short.

After the Roentgen-rays are generated, it is necessary to have some means of translating, as it were, their efficiency for the naked eye. The Roentgen-ray itself can not be distingushed by the retina. The glow of the tube, which you see, is simply the fluorescence of the tube under the electric discharge. If we place a screen coated with tungstate of calcium or barium platinum cyanide in the path of the X-ray, we find that it becomes brilliantly fluorescent; also if we bring a sensitive photographic plate or film in the vicinity of the X-ray, it is acted upon in a manner similar to the action of light. In dental work we find that the use of the fluorescent screen is of but little practical value.

The most convenient method of translating the X-ray is to use a photographic film, suitably protected from light, and placed in the oral cavity in the proper position. The films most suitable for this are the triple coated films, made by Seed & Co. While the ordinary single coated kodak film can be used, it does not give sufficient contrast. The film can be protected by being enclosed in rubber bags or they can be wrapped in heavy black paper. Where the current is heavy enough to permit of short exposures of a few seconds, the paper envelope is very convenient. The position of the film with regard to the photographic film is quite important. The film is adjusted and held in position by the finger back of the part which is desired to be radiographed. The tube should be so adjusted that the lines of maximum radiance are perpendicular to the planes of the film. Unless this point is carefully attended to, distortion will result.

The interpretation of the results obtained by the foregoing pieces of apparatus is sometimes easy and sometimes difficult. The diagnostic interpretation of a radiograph either medical, surgical or dental, should not be accepted unless made by some one who thorougly understands the principles involved. It is oftentimes very ludicrous to hear discussions concerning the so-called fallacy of the X-ray. It would be just as ludicrous to talk about the fallacy of an opera glass, microscope or chemical reaction. There are few people who have the self assurance to diagnose pathologic conditions when they look at specimens through a microscope for the first time; but the average individual feels himself perfectly confident to make a diagnosis the first time he uses the fluoroscope or the first time he sees a radiograph, and yet perhaps as much skill is required in one case as in the other.

To satisfactorily interpret a radiograph, we must first of all recollect that the radiograph is not a photograph, but a sort of shadow picture or a graphic representation of the differences of density. For example a radiograph of the hand upon which is placed a ring will show that the metal has completely shut off the X-ray, the bones have cut it off to a considerable extent and the flesh much less, while the cuff or sleeve will show as a most delicate tracery. In interpreting dental radiographs, we must remember that the enamel is less permeable than the dentin and the dentin is more opaque to the X-ray than bone, particularly the spongy bone which we find in the alveolar process. It is only by a thorough appreciation of these differences of density or permeability that the value of the radiograph can be thoroughly appreciated.

In considering the above methods of the use of the X-rays, we did not speak of the X-rays which are given by other certain newly discovered metals, radium and pollnium. These recently isolated elements present the very curious property of constantly emiting an X-ray. These rays, however, are feeble and thus far have only experimental value.

